# Epidemiology and Transmission of *Theileria orientalis* in Australasia

**DOI:** 10.3390/pathogens12101187

**Published:** 2023-09-22

**Authors:** Biniam T. Lakew, Steve Eastwood, Stephen W. Walkden-Brown

**Affiliations:** 1 Animal Science, School of Environmental and Rural Science, University of New England, Armidale, NSW 2351, Australia; bintse2@gmail.com; 2 College of Veterinary Medicine, Haramaya University, Dire Dawa P.O. Box 138, Ethiopia; 3 NSW Department of Primary Industries, Armidale, NSW 2350, Australia; steve.eastwood@dpi.nsw.gov.au

**Keywords:** *Theileria orientalis*, mechanical transmission, *Haemaphysalis longicornis*

## Abstract

Oriental theileriosis, a disease primarily impacting cattle is caused by an apicomplexan hemoprotozoan parasite, *Theileria orientalis*. It has now become established in the Australasia region. The organism was long considered a benign cause of persistent infections; however, an increase in clinical outbreaks since 2006 in the eastern Australian states and New Zealand was associated with the identification of the pathogenic Ikeda (Type 2) and Chitose (Type 1) genotypes. Unlike the pathogenic *T. parva* and *T. annulate*, which target leucocytes, clinical manifestation with *T. orientalis* is due to its effects on erythrocytes, with the infection sometimes designated as *Theileria* associated bovine anemia (TABA). In Australia and New Zealand, the tick *Haemaphysalis longicornis* is the principal vector, though other *Haemaphysalis* species are also likely vectors. The endemic status of infection with pathogenic genotypes in areas with low or absent tick populations is an apparent paradox that may be attributable to alternative modes of transmission, such as mechanical transmission by hematophagous insects (lice, mosquitoes, and biting flies), vertical transmission, and transmission via iatrogenic means. This review addresses the evidence for the different modes of transmission of *T. orientalis* with particular focus on the reported and potential vectors in Australasia.

## 1. Introduction

The evolutionary and genetic diversity of *Theileria* species [1], and in particular *Theileria orientalis*, have been reviewed [2], as have the different molecular tools and genetic markers available for the diagnosis of *T. orientalis* [3]. A recent review on integrated *T. orientalis* management, with a focus on immunity, has also been published [4]. In this review, we assess the literature related to the possible modes of transmission of the parasite in areas where ticks are both present or apparently absent.

Bovine theileriosis is a tick-borne disease caused by an intra-erythrocytic protozoan parasite of the genus *Theileria* (Apicomplexa: Piroplasmorida; Theileridae) [5]. The geographical distribution of *Theileria* species is usually restricted to tropical and subtropical regions where suitable tick vectors occur [6]. The first outbreak report of the disease was in 1902 in Africa [7]. East Coast fever (ECF), whose aetiological agent is *T. parva*, is prevalent across the eastern, central, and southern parts of Africa and was later reported in Comoros [8]. Tropical theileriosis, caused by *T. annulata* has a wider distribution than *T. Parva* [9]. Although these species are exotic to Australia and New Zealand, the countries have pathogenic strains of *T. orientalis*, resulting in significant production and productivity losses.

In Australia, a member of the *T. orientalis* complex was first detected from cattle in 1910 and was named *T. mutans* [10] due to its morphological similarity to the previously described African species [11,12]. The parasite was previously split into a three-species complex (*T. sergenti*/*buffeli*/*orientalis*), with the parasite group known as *T. buffeli* in Australia, *T. sergenti* in Japan, and *T. orientalis* in other countries. However, more recently, the organism is generally classified as *T. orientalis* based on genes encoding immunogenic piroplasm surface proteins [1]. The parasite has now become established in southeast Asia, the United States, and in Australasia, including both Australia and New Zealand.

## 2. Classification of *T. orientalis*

The Phylum Apicomplexa is divided into three orders and two suborders with the order Piroplasmorida containing the genera *Babesia* and *Theileria* [5]. *Theileria* is distinguished by sporozoite infection of leukocytes followed by maturation of schizonts into merozoites and subsequent infection of erythrocytes to form piroplasms, whereas *Babesia* infection only involves erythrocytes [13].

Globally, eight distinct genotypes of the *T. orientalis* complex, including type 1 (chitose), type 2 (Ikeda), type 3 (buffeli), and types 4–8, are currently recognized based on the sequence of the major piroplasm surface protein (MSPS) gene [1], five of which have been identified in Australia. Using the same gene, three more genotypes of *T. orientalis*, designated N-1, N-2, and N-3, have been reported to infect sheep, water buffalo, and cattle, respectively [14]. Of these 11 genotypes, only Ikeda and chitose are known to be pathogenic and cause considerable production losses, morbidity, and/or mortality in Australia and New Zealand [15,16] Whole genome sequencing showed the genetic divergence of pathogenic Ikeda compared to less pathogenic Chitose and Buffeli genotypes, and proposed the genotype as a separate species [17]. A recent study in Australia revealed the existence of genetic differences between Chitose and Buffeli [18], further strengthening the above proposition.

## 3. Epidemiology of Theileriosis Caused by *T. orientalis*

### 3.1. Worldwide Distribution

**Figure 1:**
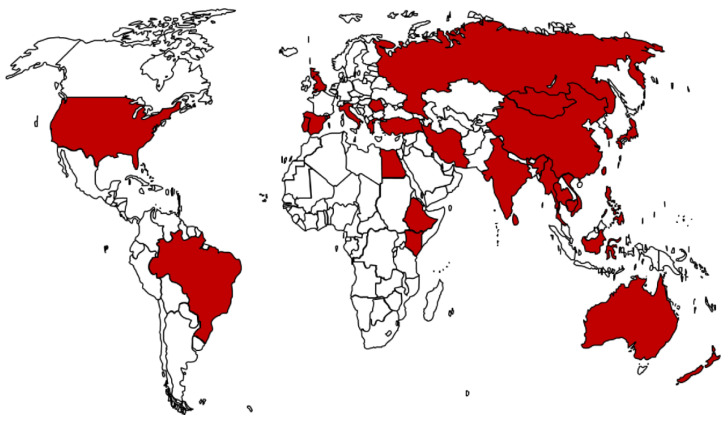
Global distribution of *T. orientalis* based on reports from different countries. Data on countries affected sourced from Yam and colleagues [2].

*Theileria* parasites infect a broad range of wild and domestic animals, with the highest prevalence in tropical and subtropical climates, and their distribution is dependent on the availability and competence of suitable tick vectors [6]. Reports on the different *T. orientalis* genotypes and affected countries have been reviewed [2] and the worldwide distribution of this parasite is shown in Figure 1.

### 3.2. Theileriosis in Australasia

#### 3.2.1. Introduction and Historical Aspects

In Australia, the infection of cattle with *T. orientalis* was considered benign with only occasional reports of clinical cases, some of which were associated with fatalities recorded in Queensland in the 1960s [19]. While *Theileria* piroplasms have been seen regularly in blood smears from cattle in NSW, until 2007–2008 it had been assumed that these were from Buffeli-type organisms, considered of low pathogenicity [20,21]. In addition, it has been suspected that Chitose-type organisms were present in Australia in the 1990s, as cattle exported from Australia that developed theileriosis within two weeks of arriving in Japan tested positive for this type [22]. While the disease has been reported in East Asia for many years, the first definitive Australian cases of clinical bovine theileriosis were registered in 2006, and were linked to the Ikeda genotype [15,23]. Since then, there has been an increase in the number of outbreaks in both beef and dairy cattle, principally in the states of NSW, QLD, Victoria [15,23], and South Australia [24] and more recently in Western Australia [25,26]. However, an infection with Ikeda does not necessarily result in clinical outbreaks, as had been shown in a recent study in which high parasitemia with the Ikeda or other *T. orientalis* genotypes was detected in apparently healthy cattle [27].

The spread of clinical theileriosis in Australia due to the genotype Ikeda has been reported to correspond with the known range of *H. longicornis* rather than with that of *H. bancrofti* or *H. humerosa* [28]. However, a study on cattle farms in the Northern tablelands of NSW reported a high prevalence of infection with the Ikeda genotype in areas where *H. longicornis* was not detected or known to be present [27]. The same study has also seen a high prevalence of infection with the Buffeli and Chitose genotypes. In Queensland, *H. bancrofti* and *H. humerosa* were shown to transmit *T. orientalis* Buffeli [29]. These findings suggest the existence of other potential biological vectors and warrants further investigation to better understand their role in the transmission of the parasite in Australia. It also raises the possibility of a role for mechanical transmission in the epidemiology of theileriosis in Australia, which will be discussed in later sections.

In New Zealand, the first published report on the *T. orientalis* genotypes (Chitose and Buffeli) was in 1982 [30] and the parasite caused sporadic clinical cases up until the emergence of Ikeda in 2012 [16,31]. Prevalence and spatial distribution studies showed that *T. orientalis* Ikeda predominantly occurs in the North Island rather than in the South Island where the distribution of *H. longicornis* is sparse [32,33]. Lawrence and colleagues [34] recently reported sheep as asymptomatic carriers for *T. orientalis* Ikeda that are capable of infecting naïve *H. longicornis*, leading to speculation that they might have played an important role in the rapid spread of oriental theileriosis in the New Zealand. Sheep may similarly have a role in the epidemiology of theileriosis in Australia and this warrants investigation.

#### 3.2.2. Pathogen Factors

In Australia, four of the *T. orientalis* genotypes—type 1 (Chitose), type 2 (Ikeda), type 3 (Buffeli), and type 5 are present [24,35,36,37]. The most important of these is the genotype Ikeda and, to a lesser extent, Chitose, which have been consistently found to be associated with clinical cases in the region. In a recent study on the Northern Tablelands of NSW, cases attributable to Ikeda were minimal despite the presence of a high infection rate with the genotype in the majority of the farms [27] and this might be due the presence of less virulent strains, as was suggested previously [38]. There was a report of a phylogenetic subgroup of Chitose, Chitose A, being strongly associated with clinical cases in Australia through mixed infections with Ikeda [38]. Moreover, the presence of the phylogenotype in a recent study was linked to a farm where high gene copies of Ikeda and ticks were detected [27]. Overall, *T. orientalis* infection often presents as a mixture of genotypes contributing to the persistence of the organism by allowing the parasite to evade the host immune response and is a clear indication of a lack of cross-protection among the genotypes [27,37,38].

The presence of Ikeda in mixed infections may result in increased virulence in some herds as it might outcompete less pathogenic genotypes, leading to their displacement by the more virulent genotype [39,40]. This was proven experimentally in cattle infected with Buffeli, Chitose, and Ikeda genotypes that become negative for the less virulent Buffeli after a few weeks [40]. A previous study on the draft genomes of Australian isolates indicated that the less pathogenic (Chitose and Buffeli) genotypes are more closely related to each other than to the pathogenic Ikeda. However, a more recent complete genomic revealed within-species differences between Chitose and Buffeli [18]. Hence, the whole genome sequencing of additional *T. orientalis* genotypes is warranted to determine whether a new species designation should be applied.

#### 3.2.3. Host Factors

Cattle infected with *T. orientalis* genotypes are regarded as a long-term carriers [41]. However, the temporal dynamics of *T. orientalis* inside the host varies depending on the genotype inoculated by ticks. An Australian study showed that the genotype Ikeda was detected first in naïve cattle introduced to endemic areas and in herds with mixed infections, suggesting it has a shorter prepatent period and/or a more efficient multiplication inside the tick vector [38]. In a different study in Australia, an analysis of clinical case records of *T. orientalis* infections from the Northern Tablelands and North Coast regions since 2009 revealed that co-infections with both pathogenic and non-pathogenic genotypes were most common [27]. Overall, the high herd and animal prevalence of infection with *T. orientalis* in healthy cattle suggests the need for caution in using *Theileria*-positive PCR results to arrive at a definitive diagnosis of clinical disease [42]. While the level of parasitemia determined by qPCR has been shown to have diagnostic value [43], this appears to have most diagnostic value at a herd rather than individual animal level [27].

A risk factor linked to the rapid spread of clinical theileriosis is the movement of infected cattle or vectors without appropriate quarantine measures [24,44]. A higher prevalence of infection in beef cattle has been postulated to be linked to more movement and exposure to ticks than dairy cattle [36], as noted in the first outbreak in Victoria [23]. However, clinical cases are not always associated with cattle introductions with clinical theileriosis reported in homebred calves in the Gloucester region of NSW [45]. The presence of wild animals known to be hosts for the different developmental stages of *H. longicornis* in Victoria and other states has also been suspected of contributing to the spread of *T. orientalis* [36,46]. Similarly, alternative mammalian hosts for *T. orientalis*, such as sheep in New Zealand, are thought to have played a significant role in the epidemiology of infection in that country [34]. The stress associated with transportation [47] and pregnancy has also been shown to be precipitating factors for clinical theileriosis [15,48].

The epidemiology of most infectious diseases changes with time after introduction, as herd immunity develops and the rates of transmission and clinical disease decline as the disease progresses from epidemic to endemic status [49]. In the Australasian literature on *T. orientalis,* it is not always clear whether the information provided relates to the epidemic or endemic phase of the disease. Furthermore, the endemic phase of piroplasm diseases such as babesiosis can typically be divided into areas of enzootic/endemic stability and those characterized by enzootic/endemic instability [50]. In the former, the conditions for transmission are good and infection rates of ticks and the mammalian host are high, but, paradoxically, disease incidence is low due to a high level of immunity in the mammalian host and the exposure of all calves to infection before 9 months of age, a period of resistance to clinical disease [51]. On the other hand, enzootic instability is characterized by conditions unfavorable for transmission with low infection rates of ticks and/or low tick infestation rates in cattle. Under these conditions, many cattle reach adulthood without exposure to piroplasms and are highly susceptible to severe clinical disease.

A similar situation has been reported for *T. orientalis* in Australia, with high levels of maternal antibody co-existing with high parasitemia loads in young calves in an area where infection is endemic, but, in this case, while calves appeared to be protected against mortality, both clinical and subclinical anemia were induced with the degree of anemia associated with the level of parasitemia [52].

#### 3.2.4. Environmental Factors

Changes in the seasonal prevalence of tick-borne pathogens are caused by changes in the abundance, infection rates, and extent of exposure to questing ticks [53]. In a study carried out in the republic of Korea (ROK), cattle grazing on a mountainous area had a statistically higher *T. orientalis* infection rate of 43% in the fall compared to 14% in summer and 11% in the spring [54]. The gradual increase in *Theileria* infection rates was caused by an increase in exposure to *H. longicornis* in the same study.

Many species of ticks are adapted to seasonal variations in climate within their geographical range. A study from Japan observed that all developmental stages of *H. longicornis*, except eggs, were able to overwinter in pasture soil, with *Theileria* being detected in the salivary glands of overwintered nymphal stages, contributing to outbreaks of epidemics in the spring [55]. In Australia and New Zealand, spring is the season for most dairy and beef calving [56] with considerable synchronicity between questing *H. longicornis* nymphs, stressed periparturient cows, and the birth of naïve calves, each contributing to outbreak episodes. There have not been equivalent studies on *H. bancrofti*, which is also a competent vector tick species.

With regard to potential mechanical animate vectors, meteorological variables, such as temperature, relative humidity, wind speed or atmospheric pressure, influence the daily activity patterns of biting flies with each species responding differently [57]. Cool temperatures limit the initiation of flight activity whereas high wind velocity disturbs the flight activity and affects the airborne olfactory cues used in host location. Tabanid activity is highly seasonal in temperate climates with high activity in the warmer months, typically summer [58,59]. The effects of meteorological variables on the daily activity patterns for Australian tabanid species suggested that tabanids in south-eastern Queensland become active between 10.00 and 15.00 h, especially on hot sunny days [60]. However, *Stomoxys* were reported to be abundant in both dry and wet seasons [61].

## 4. Lifecycle, Vectors, and Modes of Transmission

The developmental stages of *T. orientalis* pass transstadially [44,62], but attempts at demonstrating transovarian transmission have been unsuccessful [29,34]. Broadly, *Theileria* parasites are classified into two groups, transforming (*T. parva*, *T. annulate*, and *T. taurotragi*) and non-transforming (*Theileria orientalis*, *T. mutans*, and *T. velifera*), by their ability to transform leukocytes in the infected hosts [1]. In *T. parva*, there is little or no multiplication in the erythrocytes, with multiplication occurring exclusively in lymphocytes. In contrast, multiple rounds of asexual division have been observed to occur in both the erythrocytes and lymphocytes for *T. annulata* [63]. However, the major pathogenic effects of *T. orientalis* infection are associated with the destruction of infected erythrocytes and subsequent anemia. Hence, the red blood cell phase (piroplasm), rather than the leukocyte phase (schizont), drives the pathogenesis of the species [15].

### 4.1. Biological Vectors

In Australia, transmission experiments with *T. orientalis* carried out at different times, identified the ticks, *H. bancrofti* [64], *H. humerosa* (later reclassified as *H. bremneri*), and *H. longicornis* Neumann, 1901, as potential vectors [21,29,65,66]. However, the tick *H. longicornis* is the main biological vector in Australia and New Zealand [67], and in the United States [68,69]. Though their role in transmission has not yet been confirmed, several studies have detected different *T. orientalis* genotypes in a range of tick species, as is summarized in Table 1. A limitation in many of these studies is that they relied on ticks collected from hosts with results not clearly differentiating between the active infection of the tick and passive contamination via ingested host blood. The detection of theilerial DNA in questing ticks collected from pasture and, in some cases, after a long winter and before cattle were allowed to graze, is likely a better indicator of the potential role in the transmission of the parasite.

The distribution patterns of ticks in one location can vary because of fluctuations in the relative density of host animals which may sustain the life cycle and/or their introduction into non-endemic areas. In Australia, cattle are the preferred hosts of *H. longicornis* (commonly known as the “bush tick” in Australia), but sheep may also be heavily infected and have recently been implicated as asymptomatic carriers of *T. orientalis* Ikeda infections in New Zealand [34]. The Australian strain of *H. longicornis* is thought to originate from northern Japan [77]. The species is widely distributed, as far south as East Gippsland and the Murray Valley in Victoria and north to Gayndah in Queensland. It has also been isolated from inland sites such as Tenterfield and Young in New South Wales [78] and in Western Australia. While, for *H. bancrofti,* Macropodoidae (wallabies, kangaroos, and their kin) are the primary hosts, the tick has also been found on cattle [79]. There have been reports of *H. bancrofti* in the sub-coastal areas of Queensland and northern New South Wales, while it has a localized distribution in southern New South Wales [80].

**Figure 2:**
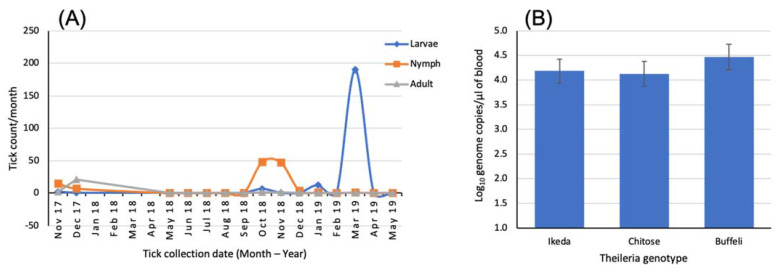
(**A**) Seasonal abundance of the different lifecycle stages of *H. bancrofti* collected from a farm in the Wollomombi area of the Northern Tablelands of NSW, Australia, and (**B**) mean *Theileria* parasitemia levels in 20 cows and heifers sampled from the same farm. Data sourced from the study of Lakew and colleagues [27].

Populations of *H. longicornis* in Australia and New Zealand are considered parthenogenic, with adult females able to lay fertile eggs in the absence of a male [79]. The eggs hatch 30–90 days after being laid with the hatched larvae questing for blood meal by climbing vertically on blades of grass to seek a host. Each engorgement occurs for 3–4 days before the tick falls to the ground and molts to the next stage. In Japan, it has been demonstrated that all developmental stages of *H. longicornis,* except the eggs, overwintered on pasture [55]. More recently, Lakew and colleagues [27] showed the seasonal dynamics of the different lifecycle stages of *H. bancrofti* on the Northern Tablelands of NSW, Australia (Figure 2).

The one host cattle tick, *Rhipicephalus microplus*, was thought to be a biological vector for *Theileria* in Australia in the first half of the 20th century, and DNA of *T. orientalis* has been detected in this tick in several recent studies. However, early transmission studies in Australia were unable to demonstrate transmission by this tick [66,81] and a similar finding has been made in a recent study in the USA [82].

### 4.2. Mechanical Vectors

Mechanical transmission involves the transfer of pathogens from an infected host or a contaminated substrate to a susceptible host, where a biological association between the pathogen and the vector is not necessary [83]. In the case of *T. orientalis*, it results in the direct transfer of haploid phase piroplasms, thereby bypassing the sexual phase of the lifecycle [38]. Many of the clinical cases between 2006 and 2010 in Australia were associated with cattle that had been moved to the coast where ticks are known to exist [15]. However, cases occur in colder and dryer inland areas where ticks were either not noted or considered rare and the prevalence of infection with *T. orientalis* can be high [27,84]. Alternative modes of transmission may thus be operating in these areas.

In Australia, there is experimental evidence demonstrating the mechanical transmission of theileria using 0.1 mL of blood from infected cattle, which had subsequently induced clinically relevant levels of *Theileria* in the recipient animal [85]. Although this mode of transmission does not appear to result in significant disease, parasitemia can persist for at least 18 months, allowing low-grade carriers to occur within herds [86]. Although passage through the tick is considered important for the ongoing fitness of the parasite, mechanical transmission could partly explain the spread of *T. orientalis* in areas where ticks are not common [27,85].

This is supported by reports of the potential for the mechanical transmission of *T. orientalis*, either through the contamination of mouthparts or the regurgitation of digestive tract contents of biting insects. Although the ixodid tick, *H. longicornis*, is the main vector for *T. orientalis*, there have been detections of the parasite in hematophagous insects including mosquitoes [87], lice [59,85,86], *Stomoxys* spp. [59,88,89], and tabanid flies [59,90]. Nevertheless, the presence of the parasite or its DNA in hematophagous insects is not surprising and in itself does not demonstrate a role in mechanical transmission.

Tabanids (commonly known as horse or march flies) are major livestock pests due to their painful bite and landing rate of up to 1000 per hour [91]. They are sexually dimorphic in feeding habit, as only females require a blood meal for ovary development. For mechanical transmission to be successful, the interruption of blood meal is crucial, resulting in the dispersal of partially fed flies to other susceptible hosts to complete the blood meal [92]. The blood meal size of tabanids ranges from 0.02 mL to 0.68 mL and, at times during peak season, can result in a loss of 200 mL of blood/host animal/day [93]. This feeding behavior makes them a likely potential vector with some species exhibiting persistent biting behavior and showing great tenacity in response to host defensive movements. A study from Queensland, Australia, observed that, among tabanid species that managed to take a blood meal, around 89% of the persistent *Pseudotabanus silvester* were successful in attaining complete engorgement on a single host, while *Tabanus pallipennis* (49%) and *Dasybasis oculata* (48%) were more sensitive to the host’s defensive movements and required as many as nine partial meals to reach engorgement [94,95]. In Australia, *T. orientalis* was not detected in tabanid flies (*Dasybasis* spp.) in one study [70]; however, a more recent study detected the parasite in *D. circumdata* [59].

Another important factor for mechanical transmission is the relative mobility of tabanid species in switching hosts after interrupted feeding since the survival of pathogens in the insect mouthparts is generally limited with time [96] although the survival time of *T. orientalis* in arthropod hosts is unknown. A study in Brazil showed that tabanid flies did transfer between horses separated by a distance of 50 m and this was recommended as a distance barrier to avoid the mechanical transmission of blood-borne parasites [97]. An understanding of the interaction between different tabanid species and cattle is important in predicting the likely role in mechanical transmission of *T. orientalis*, followed by critical experimentation to demonstrate transmission.

In addition to mechanical transmission via the contaminated mouthparts of biting flies, there is experimental evidence showing that stable flies (*Stomoxys* spp.) can regurgitate part of their previous blood meal before taking up another, resulting in the transfer of high doses of pathogens [89]. Blood can remain for 24 h or more in the crop before being directed either to the gut or partially regurgitated during the early stage of a new blood meal. In such conditions, a partial regurgitation of blood from the crop would allow a delayed transmission, possibly up to 24 h or more. In tabanids, the interval between blood meals is quite long (5–7 days), above the maximum survival of most pathogens. However, in stomoxyine flies, which are frequent feeders the interval between blood meals is variable, from 4 to 72 h [98].

In the stable fly, both males and females are blood feeders, with females typically ingesting blood meals an average of 1.8 times per day, and males 2.8 times per day, with an average volume of 11–15 µL of blood per meal [99]. It has been shown that male stable flies carried significantly higher piroplasm DNA compared to females [89]. The buffalo fly, *Haematobia irritans exigua*, which is a close relative of the stable fly, feeds 10–40 times a day. In Australia, theilerial DNA has been detected in buffalo flies [59] and, taking into account their swarming behavior when feeding on cattle, this makes them a candidate for mechanical transmission. Overall, the likelihood for mechanical transmission depends on the volume of blood left on the mouthparts after feeding, the density of biting insects feeding on the host, the level of parasitemia in the host blood, and the relative proportion of infected and non-infected hosts which are close together.

There is experimental evidence of *Linognathus vituli* transmission of *Theileria orientalis* to splenectomized calves 49 days after feeding on infected cattle that had been inoculated with a tick-derived sporozoite suspension [86]. Lice are the most common ectoparasites of cattle in the temperate zones of Australia [100], with *Linognathus vituli* found mainly on dairy cattle and *Haematopinus eurysternus* on calves [101]. There are six species of cattle lice and reports of the detection of theilerial DNA have been made in the long-nosed cattle louse (*Linognathus vituli*) [59,85] and recently in the short-nosed cattle louse (*Haematopinus eurysternus*) [59]. The mechanical transmission of *T. orientalis* by lice is a concern as lice become more prevalent during winter when cattle experience physiological and nutritional stresses, both of which have been considered risk factors for oriental theileriosis. A study from Japan demonstrated that infestation with lice was responsible for the high rate of infection with *T. orientalis* in cattle during winter and among in-house cattle where ticks and horse flies were not active [86]. Nevertheless, information on the frequency of movement of sucking lice between hosts is scarce and is essential to quantify their role in the epidemiology of bovine theileriosis in Australia and overseas.

Biting midges, *Culicoides* (Diptera: Ceratopogonidae), are among the smallest hematophagous flies, measuring 1–5 mm in length, with only females seeking blood for egg development [102]. They cause annoyance with high biting intensity with the average size of a blood meal ranging between 0.01 µL and 0.06 µL [103]. The *Culicoides* species, *C. brevitarsis*, *C. marksi*, *C. dycei*, *C. victoriae*, *C. schultzei*, and *C. peregrinus*, are considered the most important species feeding on cattle [104]. As artificial infection in susceptible calves by subcutaneous inoculation with a suspension of the parasite was possible [55], the pool feeding nature of *Culicoides* [103] also makes them potential vectors. Moreover, a recent study detected the DNA of *T. orientalis* in *C. brevitarsis* and *C. victoriae* [59]. Of particular importance is *C. brevitarsis*, which has been previously reported to have seasonal movement from coastal areas, where the parasite is endemic, to inland areas [105], greatly increasing the risk of introduction of the parasite to non-endemic areas.

Overall, mechanical transmission results in the absence of sexual reproduction of the parasite and is expected to reduce genetic diversity within the parasite population [89]. Hence, the extensive mechanical transfer of the parasite would be expected to decrease the ability of the parasite to evade the host immune system and this method of spread is thought to not be important in the wider epidemiology of the disease [106]. However, this mode of transmission is enhanced by the long-term persistence of theileriae in the blood stream. Furthermore, mechanically transferred parasitoses have been shown to produce viable infections in ticks, which subsequently developed sporozoites in their salivary glands after feeding on an inoculated calf [86].

### 4.3. Other Means of Transmission

Given the small volume of blood required for the transmission of *T. orientalis*, other modes of transmission need to be considered. One such method is vertical transmission between dams and their calves. In Australia, the microscopic detection of *Theileria* piroplasms was achieved from a 4-day-old calf [45] and using qPCR from both a newborn and fetal calf [52,107], which is indicative of transplacental transmission, though it was suggested to play a minor role in the transmission of *T. orientalis*. Similar studies in New Zealand and Japan have also shown that the vertical transplacental transmission of *T. orientalis* (Ikeda) infection is unlikely in chronically infected dairy cows [108,109]. In the latter study, infection with *T. orientalis* was not detected in calves born to infected dams 0–30 days post-delivery. However, by 4 months of age, 88% of the calves had become positive for the Ikeda genotype despite the presence of mixed infection in some of the dams. These results suggest that, in a low-risk tick-infested area, the vertical transmission of *T. orientalis* could take at least three months to become detectable by PCR.

It has also been shown that *T. orientalis* can be transmitted by low numbers of piroplasms, something that might also occur iatrogenically [85]. Routine husbandry practices that potentially result in the transfer of piroplasms include re-using needles between cattle, contaminated castration knives and dehorning equipment, and ear marking pliers. The risk of such iatrogenic transmission would depend on the volume and parasitemia of the blood transferred and on the ability of the parasite to survive outside a host before being inoculated into a susceptible animal. The latter is a gap in the knowledge relevant to all forms of mechanical transmission.

Other potential means of infection in homebred cattle include the infection of calves through blood transfer in the colostrum [85]. It has been demonstrated that leukocytes were able to transfer through the colostrum into the neonatal bloodstream [110,111], implying that infected erythrocytes could possibly transfer into a calf within 24 h of birth, releasing piroplasms. Although the capacity for ingested piroplasms to transfer into recipient erythrocytes is not known, macroschizonts of *T. parva* within cultured lymphoblasts were transferred into recipient lymphocytes when inoculated into recipients [112]. In a study from Australia, where 13.3% of the dams contained clinically significant quantities of *T. orientalis* piroplasms in the colostrum, calves receiving colostrum within 6 h of birth failed to become qPCR positive [85], though the number of colostrum samples used in the experiment were insufficient to exclude the possibility of the colostral transmission of piroplasms.

Antibodies are thought to have little role in immunity to theileria infections, since the main mechanism of immune protection against intracellular parasites is cell-mediated [113]. The epitheliochorial placentation in cattle precludes the prenatal transfer of maternal antibodies from cow to calf which are, instead, acquired from the colostrum. In a study on the transplacental transmission of *T. orientalis*, it was unclear whether seropositivity and a moderate infection intensity, as demonstrated by qPCR in a calf 36 h post-birth, were due to antibodies from a fetal immune response to infection in utero or were maternally derived post-partum [52]. Nonetheless, the high seropositive status in calves in this study was attributed to high concentrations of antibodies in the colostrum. Whether antibodies are protective against *T. orientalis* is currently unclear; however, the peak in parasite load in all calves tested in the same study occurred after the decline in antibody levels, at 5–8 weeks post-partum, suggestive of some protective effect.

## 5. Conclusions

In Australia, the benign Buffeli genotype of *T. orientalis* has long been considered to cause a mild persistent infection. However, since 2006, an increase in outbreaks of clinical theileriosis has been associated with the newly identified Ikeda and Chitose genotypes, particularly the former, and these are now becoming established in Australia and New Zealand. Moreover, there are reports of variation in virulence within the various *T. orientalis* genotypes. Although the ixodid tick, *H. longicornis*, is the known biological vector in Australasia, there is indirect evidence of transmission by other tick species. Nevertheless, *Theileria* infection is reported in areas where ticks are not detected. Under such circumstances, the mechanical transmission of *T. orientalis* by hematophagous insects, especially by sucking lice and stomoxyine flies [59], may play a role in transmission but, to date, this has only been demonstrated in the case of sucking lice. Mechanical transmission by other means, including husbandry practices involving the transfer of blood [86] and transplacental transfer [52,108], are likely to contribute to transmission, while vertical transmission through oral consumption of the colostrum is unlikely [85,109].

## Figures and Tables

**Table 1:** The different *T. orientalis* MPSP genotypes detected in the likely potential vector/s in the world.

Country	Tick Species	Source of Ticks	*T. orientalis* MPSP Genotypes Detected	References
Australia	*H. longicornis*	engorged ticks	Type 1, 2, and 3	[27,70]
	*H. bancrofti*	questing ticks	Type 1 and 3	
Japan	*H. megaspinosa,*	questing ticks	Type 1 and 2	[71]
	*H. douglasi,*			
	*Ixodes persulcatus,*			
	*I. ovatus*			
Mongolia	*Dermacentor nuttalli*	engorged ticks	Types 1, 3, 5, 7, and N-3	[72]
Ethiopia	*R. evertsi evertsi*	engorged ticks	Type 3	[73,74]
	*R. decoloratus*			
	*Rh. praetextatus*			
Turkey	*Hyalomma excavatum Boophilus annulatus*	engorged ticks	Type 3	[75]
Vietnam	*Boophilus microplus*	engorged ticks	Types 5 and 7	[14]
China	*R. microplus*	engorged ticks and questing ticks	Types 1–5, 7, and N3	[76]

## References

[1] Sivakumar T., Hayashida K., Sugimoto C., Yokoyama N. (2014). Evolution and genetic diversity of Theileria. Infect. Genet. Evol..

[2] Yam J., Bogema D., Jenkins C. (2018). Oriental Theileriosis. Ticks and Tick-Borne Pathogens.

[3] Gebrekidan H., Perera P.K., Ghafar A., Abbas T., Gasser R.B., Jabbar A. (2019). An appraisal of oriental theileriosis and the *Theileria orientalis* complex, with an emphasis on diagnosis and genetic characterisation. Parasitol. Res..

[4] Emery D.L. (2021). Approaches to Integrated Parasite Management (IPM) for *Theileria orientalis* with an Emphasis on Immunity. Pathogens.

[5] Taylor M.A., Coop R.L., Wall R.L. (2016). Veterinary Parasitology.

[6] Jabbar A., Abbas T., Sandhu Z.-U., A Saddiqi H., Qamar M.F., Gasser R.B. (2015). Tick-borne diseases of bovines in Pakistan: Major scope for future research and improved control. Parasites Vectors.

[7] Robertson W. (1904). African coast fever. J. Comp. Pathol. Ther..

[8] Deken R.D., Martin V., Saido A., Madder M., Brandt J., Geysen D. (2007). An outbreak of East Coast Fever on the Comoros: A consequence of the import of immunised cattle from Tanzania?. Vet. Parasitol..

[9] Gul N., Ayaz S., Gul I., Adnan M., Shams S., Akbar N. (2015). Tropical Theileriosis and East Coast Fever in Cattle: Present, Past and Future Perspective. Int. J. Curr. Microbiol. App. Sci..

[10] Dodd S. (1910). Piroplasmosis of cattle in Queensland. J. Comp. Pathol. Ther..

[11] Seddon H. (1952). Diseases of Domestic Animals in Australia: Pt. 4.

[12] Stewart N., Standfast N., Baldock F., Reid D., Vos A. (1992). The distribution and prevalence of Theileria buffeli in cattle in Queensland. Aust. Vet. J..

[13] Uilenberg G. (2006). Babesia—A historical overview. Vet. Parasitol..

[14] Khukhuu A., Lan D.T.B., Long P.T., Ueno A., Li Y., Luo Y., de Macedo A.C.C., Matsumoto K., Inokuma H., Kawazu S.-I. (2011). Molecular Epidemiological Survey of *Theileria orientalis* in Thua Thien Hue Province, Vietnam. J. Vet. Med. Sci..

[15] Izzo M., Poe I., Horadagoda N., De Vos A., House J. (2010). Haemolytic anaemia in cattle in NSW associated with *Theileria* infections. Aust. Vet. J..

[16] McFadden A., Rawdon T.G., Meyer J., Makin J., Morley C.M., Clough R.R., Tham K., Müllner P., Geysen D. (2011). An outbreak of haemolytic anaemia associated with infection of *Theileria orientalis* in naive cattle. N. Z. Vet. J..

[17] Bogema D.R., Micallef M.L., Liu M., Padula M.P., Djordjevic S.P., Darling A.E., Jenkins C. (2018). Analysis of *Theileria orientalis* draft genome sequences reveals potential species-level divergence of the Ikeda, Chitose and Buffeli genotypes. BMC Genom..

[18] Yam J., Bogema D.R., Micallef M.L., Djordjevic S.P., Jenkins C. (2022). Complete Genomes of *Theileria orientalis* Chitose and Buffeli Genotypes Reveal within Species Translocations and Differences in ABC Transporter Content. Pathogens.

[19] Rogers R.J., Callow L.L. (1966). Three fatal cases of theileria mutans infection. Aust. Vet. J..

[20] Callow L.L. (1984). Animal Health in Australia. Volume 5. Protozoal and Rickettsial Diseases.

[21] Stewart N.P., de Vos A.J., McGregor W., Shiels I. (1987). Haemaphysalis humerosa not H. longicornis is the likely vector of Theileria buffeli in Australia. Aust. Vet. J..

[22] Kubota S., Sugimoto C., Kakuda T., Onuma M. (1996). Analysis of immunodominant piroplasm surface antigen alleles in mixed populations of Theileria sergenti and T. buffeli. Int. J. Parasitol..

[23] Islam M.K., Jabbar A., Campbell B.E., Cantacessi C., Gasser R.B. (2011). Bovine theileriosis–An emerging problem in south-eastern Australia?. Infect. Genet. Evol..

[24] Gebrekidan H., Gasser R.B., Perera P.K., McGrath S., McGrath S., Stevenson M.A., Jabbar A. (2015). Investigating the first outbreak of oriental theileriosis in cattle in South Australia using multiplexed tandem PCR (MT-PCR). Ticks Tick-Borne Dis..

[25] Forshaw D., Alex S.M., Palmer D.G., Cotter J., Roberts W.D., Jenkins C., Hair S. (2020). *Theileria orientalis* Ikeda genotype infection associated with anaemia, abortion and death in beef cattle in Western Australia. Aust. Vet. J..

[26] Leong C.-C., Oskam C.L., Barbosa A.D., Aleri J.W. (2023). Distribution and Prevalence of *Theileria orientalis* Genotypes in Adult Lactating Dairy Cows in South West Region of Western Australia. Pathogens.

[27] Lakew B.T., Kheravii S.K., Wu S.-B., Eastwood S., Andrew N.R., Jenkins C., Walkden-Brown S.W. (2020). Endemic infection of cattle with multiple genotypes of *Theileria orientalis* on the Northern Tablelands of New South Wales despite limited presence of ticks. Ticks Tick-Borne Dis..

[28] Jenkins C. (2018). Bovine theileriosis in Australia: A decade of disease. Microbiol. Aust..

[29] Stewart N.P., Vos A.J., Shiels I., McGregor W. (1987). The experimental transmission of Theileria buffeli of cattle in Australia by Haemaphysalis humerosa. Aust. Vet. J..

[30] James M., Saunders B.W., Guy L.A., Brookbanks E.O., Charleston W.A.G., Uilenberg G. (1984). *Theileria orientalis*, a blood parasite of cattle. First report in New Zealand. N. Z. Vet. J..

[31] Lawrence K., McFadden A., Gias E., Pulford D., Pomroy W. (2015). Epidemiology of the epidemic of bovine anaemia associated with *Theileria orientalis* (Ikeda) between August 2012 and March 2014. N. Z. Vet. J..

[32] McFadden A., Gias E., Heuer C., McFadden F.S., Pulford D. (2016). Prevalence and spatial distribution of cattle herds infected with *Theileria orientalis* in New Zealand between 2012 and 2013. N. Z. Vet. J..

[33] Heath A. (2016). Biology, ecology and distribution of the tick, *Haemaphysalis longicornis* Neumann (Acari: Ixodidae) in New Zealand. N. Z. Vet. J..

[34] Lawrence K., Gedye K., Hickson R., Wang B., Carvalho L., Zhao Y., Pomroy W. (2021). The role of sheep (Ovis aries) in maintaining *Theileria orientalis* Ikeda type infection. Vet. Parasitol..

[35] Perera P.K., Gasser R.B., Read E., Malmo J., Nguyen H., Nyein S., Cheng A., Jex A.R., Rawlin G., Spithill T.W. (2015). Use of multiplexed tandem PCR to estimate the prevalence and intensity of *Theileria orientalis* infections in cattle. Infect. Genet. Evol..

[36] Perera P.K., Gasser R.B., Anderson G.A., Jeffers M., Bell C.M., Jabbar A. (2013). Epidemiological survey following oriental theileriosis outbreaks in Victoria, Australia, on selected cattle farms. Vet. Parasitol..

[37] Kamau J., de Vos A.J., Playford M., Salim B., Kinyanjui P., Sugimoto C. (2011). Emergence of new types of *Theileria orientalis* in Australian cattle and possible cause of theileriosis outbreaks. Parasites Vectors.

[38] Jenkins C., Micallef M., Alex S., Collins D., Djordjevic S., Bogema D. (2015). Temporal dynamics and subpopulation analysis of *Theileria orientalis* genotypes in cattle. Infect. Genet. Evol..

[39] Eamens G.J., Bailey G., Jenkins C., Gonsalves J.R. (2013). Significance of *Theileria orientalis* types in individual affected beef herds in New South Wales based on clinical, smear and PCR findings. Vet. Parasitol..

[40] Kamau J., Salim B., Yokoyama N., Kinyanjui P., Sugimoto C. (2011). Rapid discrimination and quantification of *Theileria orientalis* types using ribosomal DNA internal transcribed spacers. Infect. Genet. Evol..

[41] Kubota S., Sugimoto C., Onuma M. (1996). Population dynamics of *Theileria sergenti* in persistently infected cattle and vector ticks analysed by a polymerase chain reaction. Parasitology.

[42] Proctor A., Ball M., Freeman P., Jenkins C., Bogema D.R. (2016). Prevalence of *Theileria orientalis* types in beef cattle herds on the North Coast of New South Wales. Aust. Vet. J..

[43] Bogema D.R., Deutscher A.T., Fell S., Collins D., Eamens G.J., Jenkins C. (2015). Development and Validation of a Quantitative PCR Assay Using Multiplexed Hydrolysis Probes for Detection and Quantification of *Theileria orientalis* Isolates and Differentiation of Clinically Relevant Subtypes. J. Clin. Microbiol..

[44] Gachohi J., Skilton R., Hansen F., Ngumi P., Kitala P. (2012). Epidemiology of East Coast fever (Theileria parva infection) in Kenya: Past, present and the future. Parasites Vectors.

[45] Swilks E., Jenkins C., Poynting A., Collins D., Krebs G. (2017). Prevalence and effect of *Theileria orientalis* infection in homebred calves in the Gloucester region of New South Wales, Australia. Aust. Vet. J..

[46] Storey-Lewis B., Mitrovic A., McParland B. (2018). Molecular detection and characterisation of Babesia and Theileria in Australian hard ticks. Ticks Tick-Borne Dis..

[47] Gebrekidan H., Nelson L., Smith G., Gasser R.B., Jabbar A. (2016). An outbreak of oriental theileriosis in dairy cattle imported to Vietnam from Australia. Parasitology.

[48] Watts J., Playford M., Hickey K. (2016). *Theileria orientalis*: A review. N. Z. Vet. J..

[49] Thrusfield M. (2018). Veterinary Epidemiology.

[50] Mahoney D.F., Ross D.R. (1972). Epizootiological factors in the control of bovine babesiosis. Aust. Vet. J..

[51] Mahoney D., Wright I.G., Goodger B.V., Mirre G.B., Sutherst R.W., Utech K.B.W. (1981). The transmission ofbabesia bovisin herds of european and zebu x european cattle infested with the tick, boophilus microplus. Aust. Vet. J..

[52] Swilks E., Fell S.A., Hammer J.F., Sales N., Krebs G.L., Jenkins C. (2017). Transplacental transmission of *Theileria orientalis* occurs at a low rate in field-affected cattle: Infection in utero does not appear to be a major cause of abortion. Parasites Vectors.

[53] Randolph S.E. (2009). Chapter 6 Epidemiological Consequences of the Ecological Physiology of Ticks. Adv. Insect Physiol..

[54] Choi K.-S., Yu D.-H., Chae J.-S., Park B.-K., Yoo J.-G., Park J. (2016). Seasonal changes in hemograms and *Theileria orientalis* infection rates among Holstein cattle pastured in the mountains in the Republic of Korea. Prev. Vet. Med..

[55] Fujisaki K., Ito Y., Kamio T., Kitaoka S. (1985). The presence of *Theileria sergenti* in *Haemaphysalis longicornis* overwintering in pasture in Japan. Ann. Trop. Med. Parasitol..

[56] McFadden A., Heuer C., Jackson R., West D., Parkinson T. (2005). Reproductive performance of beef cow herds in New Zealand. N. Z. Vet. J..

[57] VAN Hennekeler K., Jones R.E., Skerratt L.F., Muzari M.O., Fitzpatrick L.A. (2011). Meteorological effects on the daily activity patterns of tabanid biting flies in northern Queensland, Australia. Med. Vet. Entomol..

[58] Baldacchino F., Desquesnes M., Mihok S., Foil L.D., Duvallet G., Jittapalapong S. (2014). Tabanids: Neglected subjects of research, but important vectors of disease agents!. Infect. Genet. Evol..

[59] Lakew B.T., Kheravii S.K., Wu S.-B., Eastwood S., Andrew N.R., Nicholas A.H., Walkden-Brown S.W. (2021). Detection and distribution of haematophagous flies and lice on cattle farms and potential role in the transmission of *Theileria orientalis*. Vet. Parasitol..

[60] Spratt D. (1974). Comparative epidemiology of Dirofilaria roemeri infection in two regions of Queensland. Int. J. Parasitol..

[61] Ahmed A., Okiwelu S., Samdi S. (2006). Species diversity, abundance and seasonal occurrence of some biting flies in Southern Kaduna, Nigeria. Afr. J. Biomed. Res..

[62] Emery D., Burgh S.D., Dinh T.H.H.H., Rolls P., Carter P. (2020). Merozoites of *Theileria Orientalis* Buffeli Reduce Parasitosis Following Challenge by Ticks Infested with T. Orientalis Ikeda. Res. Sq..

[63] Shaw M.K. (2002). Theileria Development and Host Cell Invasion.

[64] Nuttall G.H.F., Warburton C., Cooper W.F., Robinson L.E. (1915). Ticks: A Monograph of the Ixodidea Part III. The Genus Haemaphysalis.

[65] Stewart N., Devos A., Shiels I., Jorgensen W. (1989). Transmission of Theileria buffeli to cattle by Haemaphysalis bancrofti fed on Artificially Infected Mice. Vet. Parasitol..

[66] Riek R.F. (1982). Epidemiology and transmission of Theileria sp of cattle in Australia. Aust. Vet. J..

[67] Marendy D., Baker K., Emery D., Rolls P., Stutchbury R. (2019). *Haemaphysalis longicornis*: The life-cycle on dogs and cattle, with confirmation of its vector status for *Theileria orientalis* in Australia. Vet. Parasitol..

[68] Dinkel K.D., Herndon D.R., Noh S.M., Lahmers K.K., Todd S.M., Ueti M.W., Scoles G.A., Mason K.L., Fry L.M. (2021). A U.S. isolate of *Theileria orientalis*, Ikeda genotype, is transmitted to cattle by the invasive Asian longhorned tick, *Haemaphysalis longicornis*. Parasites Vectors.

[69] Thompson A.T., White S., Shaw D., Egizi A., Lahmers K., Ruder M.G., Yabsley M.J. (2020). *Theileria orientalis* Ikeda in host-seeking *Haemaphysalis longicornis* in Virginia, U.S.A. Ticks Tick-Borne Dis..

[70] Hammer J.F., Emery D., Bogema D.R., Jenkins C. (2015). Detection of *Theileria orientalis* genotypes in *Haemaphysalis longicornis* ticks from southern Australia. Parasites Vectors.

[71] Yokoyama N., Sivakumar T., Ota N., Igarashi I., Nakamura Y., Yamashina H., Matsui S., Fukumoto N., Hata H., Kondo S. (2012). Genetic diversity of *Theileria orientalis* in tick vectors detected in Hokkaido and Okinawa, Japan. Infect. Genet. Evol..

[72] Altangerel K., Battsetseg B., Battur B., Sivakumar T., Batmagnai E., Javkhlan G., Tuvshintulga B., Igarashi I., Matsumoto K., Inokuma H. (2011). The first survey of *Theileria orientalis* infection in Mongolian cattle. Vet. Parasitol..

[73] Kumsa B., Signorini M., Teshale S., Tessarin C., Duguma R., Ayana D., Martini M., Cassini R. (2013). Molecular detection of piroplasms in ixodid ticks infesting cattle and sheep in western Oromia, Ethiopia. Trop. Anim. Health Prod..

[74] Hornok S., Abichu G., Meli M.L., Tánczos B., Sulyok K.M., Gyuranecz M., Gönczi E., Farkas R., Hofmann-Lehmann R. (2014). Influence of the Biotope on the Tick Infestation of Cattle and on the Tick-Borne Pathogen Repertoire of Cattle Ticks in Ethiopia. PLoS ONE.

[75] Aktas M., Altay K., Ozubek S., Dumanli N. (2012). A survey of ixodid ticks feeding on cattle and prevalence of tick-borne pathogens in the Black Sea region of Turkey. Vet. Parasitol..

[76] Li L.-H., Wang J.-Z., Zhu D., Li X.-S., Lu Y., Yin S.-Q., Li S.-G., Zhang Y., Zhou X.-N. (2020). Detection of novel piroplasmid species and Babesia microti and *Theileria orientalis* genotypes in hard ticks from Tengchong County, Southwest China. Parasitol. Res..

[77] Hoogstraal H., Roberts F.H.S., Kohls G.M., Tipton V.J. (1968). Review of Haemaphysalis (Kaiseriana) longicornis Neumann (Resurrected) of Australia, New Zealand, New Caledonia, Fiji, Japan, Korea, and Northeastern China and USSR, and Its Parthenogenetic and Bisexual Populations (Ixodoidea, Ixodidae). J. Parasitol..

[78] Roberts F. (1963). A systematic study of the Australian species of the genus Haemaphysalis Koch (Acarina: Ixodidae). Aust. J. Zool..

[79] Roberts F.H.S. (1970). Australian Ticks.

[80] Laan B., Handasyde K., Beveridge I. (2011). Occurrence of the tick Haemaphysalis bancrofti Nuttall & Warburton, 1915 in Victoria with additional data on its distribution and with scanning electron micrographs of life cycle stages. Proc. R. Soc. Vic..

[81] Callow L.L., Hoyte H.M.D. (1961). Transmission experiments using *Babesia bigemina, Theileria mutans*, *Borrelia* sp.; the cattle tick. Aust. Vet. J..

[82] Onzere C.K., Herndon D.R., Hassan A., Oyen K., Poh K.C., Scoles G.A., Fry L.M. (2023). A U.S. Isolate of *Theileria orientalis* Ikeda Is Not Transstadially Transmitted to Cattle by *Rhipicephalus microplus*. Pathogens.

[83] Foil L.D., Gorham J.R. (2004). Mechanical Transmission of Disease Agents by Arthropods, in Medical Entomology.

[84] Biddle A., Eastwood S., Martin L., Freeman P., Druce E. (2013). A survey to determine the prevalence of *Theileria* spp. in beef cattle in the northern tablelands of New South Wales. Aust. Vet. J..

[85] Hammer J.F., Jenkins C., Bogema D., Emery D. (2016). Mechanical transfer of *Theileria orientalis*: Possible roles of biting arthropods, colostrum and husbandry practices in disease transmission. Parasites Vectors.

[86] Fujisaki K., Kamio T., Kawazu S., Shimizu S., Shimura K. (1993). *Theileria sergenti*: Experimental transmission by the long-nosed cattle louse, *Linognathus vituli*. Ann. Trop. Med. Parasitol..

[87] De Marco M.F., Brugman V., Hernández-Triana L., Thorne L., Phipps L., Nikolova N., Fooks A., Johnson N. (2016). Detection of *Theileria orientalis* in mosquito blood meals in the United Kingdom. Vet. Parasitol..

[88] Changbunjong T., Sungpradit S., Kanthasaewee O., Sedwisai P., Tangsudjai S., Ruangsittichai J. (2016). Molecular Detection of Theileria and Babesia in a Diversity of Stomoxyini Flies (Diptera: Muscidae) from Khao Yai National Park, Thailand. Thai J. Vet. Med..

[89] Hornok S., Takács N., Szekeres S., Szőke K., Kontschán J., Horváth G., Sugár L. (2020). DNA of *Theileria orientalis*, *T. equi* and *T. capreoli* in stable flies (*Stomoxys calcitrans*). Parasites Vectors.

[90] Jirapattharasate C., Changbunjong T., Sedwisai P., Weluwanarak T. (2018). Molecular detection of piroplasms in haematophagus flies in the Nakhon Pathom and Kanchanaburi Provinces, Thailand. Vet. Integr. Sci..

[91] Foil L.C., Foil C.S. (1988). Dipteran parasites of horses. Equine Pract..

[92] Foil L., Stage D., Adams W.V., Issel C.J. (1985). Observations of tabanid feeding on mares and foals. Am. J. Vet. Res..

[93] Hollander A.L., Wright R.E. (1980). Impact of Tahanids on Cattle: Blood Meal Size and Preferred Feeding Sites. J. Econ. Entomol..

[94] Muzari M., Jones R., Skerratt L., Duran T. (2010). Feeding success and trappability of horse flies evaluated with electrocuting nets and odour-baited traps. Vet. Parasitol..

[95] Muzari M., Skerratt L., Jones R., Duran T. (2010). Alighting and feeding behaviour of tabanid flies on horses, kangaroos and pigs. Vet. Parasitol..

[96] Foil L. (1989). Tabanids as vectors of disease agents. Parasitol. Today.

[97] Barros A., Foil L. (2007). The influence of distance on movement of tabanids (Diptera: Tabanidae) between horses. Vet. Parasitol..

[98] Foil L., Hogsette J. (1994). Biology and control of tabanids, stable flies and horn flies. Rev. Sci. Tech.-Off. Int. Des Épizooties.

[99] Schowalter T., Klowden M. (1979). Blood Meal Size of the Stable Fly, Stomoxys Calcitrans, Measured by the HiCN [Hemoglobin-Cyanide] Method. Mosq. News.

[100] Holdsworth P.A. (2002). Use of Macrocyclic Lactones to Control Cattle Parasites in Australia and New Zealand.

[101] Bailey G. (2015). Cattle Lice, Primefact 337.

[102] Yu Y., Liu J., Liu G., Liu Z., Hao B., Yan G., Zhao T. (2005). Ceratopogonidae of China. Insecta Diptera.

[103] Venter G., Hamblin C., Paweska J. (2003). Determination of the oral susceptibility of South African livestock-associated biting midges, Culicoides species, to bovine ephemeral fever virus. Med. Vet. Entomol..

[104] Standfast H.A., Dyce A. (1972). Potential vectors of arboviruses of cattle and buffalo in Australia. Aust. Vet. J..

[105] Bishop A.L., Barchia I.M., Spohr L.J. (2000). Models for the dispersal in Australia of the arbovirus vector, Culicoides brevitarsis Kieffer (Diptera: Ceratopogonidae). Prev. Vet. Med..

[106] Lawrence K., Gedye K., Pomroy W. (2019). A longitudinal study of the effect of *Theileria orientalis* Ikeda type infection on three New Zealand dairy farms naturally infected at pasture. Vet. Parasitol..

[107] Baek B.K., Soo K.B., Kim J.H., Hur J., O Lee B., Jung J.M., Onuma M., O Oluoch A., Kim C.-H., Kakoma I. (2003). Verification by polymerase chain reaction of vertical transmission of *Theileria sergenti* in cows. Can. J. Vet. Res. = Rev. Can. Rech. Vet..

[108] Lawrence K., Gedye K., McFadden A., Pulford D., Pomroy W. (2016). An observational study of the vertical transmission of *Theileria orientalis* (Ikeda) in a New Zealand pastoral dairy herd. Vet. Parasitol..

[109] Mekata H., Minamino T., Mikurino Y., Yamamoto M., Yoshida A., Nonaka N., Horii Y. (2018). Evaluation of the natural vertical transmission of *Theileria orientalis*. Vet. Parasitol..

[110] Reber A., Donovan D., Gabbard J., Galland K., Aceves-Avila M., Holbert K., Marshall L., Hurley D. (2008). Transfer of maternal colostral leukocytes promotes development of the neonatal immune system: Part II. Effects on neonatal lymphocytes. Vet. Immunol. Immunopathol..

[111] Donovan D.C., Reber A.J., Gabbard J.D., Aceves-Avila M., Galland K.L., Holbert K.A., Ely L.O., Hurley D.J. (2007). Effect of maternal cells transferred with colostrum on cellular responses to pathogen antigens in neonatal calves. Am. J. Vet. Res..

[112] Emery D.L., I Morrison W., Buscher G., Nelson R.T. (1982). Generation of cell-mediated cytotoxicity to Theileria parva (East Coast fever) after inoculation of cattle with parasitized lymphoblasts. Pediatrics.

[113] Goddeeris B.M., Morrison W.I. (1994). Cell-Mediated Immunity in Ruminants.

